# Protocol to perform cell-type-specific transcriptome-wide association study using scPrediXcan framework

**DOI:** 10.1016/j.xpro.2025.104306

**Published:** 2026-02-13

**Authors:** Yichao Zhou, Sarah Sumner, Temidayo Adeluwa, Lisha Zhu, Sofia Salazar-Magaña, Hyunki Kim, Saideep Gona, Festus Nyasimi, Rohit Kulkarni, Joseph Powell, Ravi Madduri, Boxiang Liu, Mengjie Chen, Hae Kyung Im

**Affiliations:** 1 Committee of Genetic, Genomics, and Systems Biology, University of Chicago, Chicago, IL, USA; 2 Department of Medicine, Section of Genetic Medicine, University of Chicago, Chicago, IL, USA; 3 Department of Medicine, Harvard Medical School, Boston, MA, USA; 4 Department of Human Genetics, University of Chicago, Chicago, IL, USA; 5 UNSW Cellular Genomics Futures Institute, University of New South Wales, Sydney, NSW, Australia; 6 Translational Genomics, Garvan Institute of Medical Research, Sydney, NSW 2010, Australia; 7 Data Science and Learning Division, Argonne National Laboratory, Chicago, IL, USA; 8 Department of Pharmacy and Pharmaceutical Sciences, National University of Singapore, Singapore, Singapore

**Keywords:** Bioinformatics, sequence analysis, Genetics, genomics, gene expression

## Abstract

The scPrediXcan framework enables cell-type-specific transcriptome-wide association studies (TWASs) by integrating deep learning-based prediction of gene expression from DNA sequence and epigenetic features. We present a protocol for scPrediXcan: training cell-type-specific models for expression prediction, predicting personalized expression, and testing associations with genome-wide association study (GWAS) summary statistics. This framework produces scalable TWAS models for different cellular contexts with minimal computational burden.

For complete details on the use and execution of this protocol, please refer to Zhou et al.^1^

## Highlights

• Perform cell-type-specific TWASs with scPrediXcan using single-cell data
• Train gene expression models for various cell types without specialized hardware
• Prioritize causal genes for diseases across cellular contexts

## Abstract (editor highlights)

Publisher’s note: Undertaking any experimental protocol requires adherence to local institutional guidelines for laboratory safety and ethics.

## Graphical abstract

**Figure:**
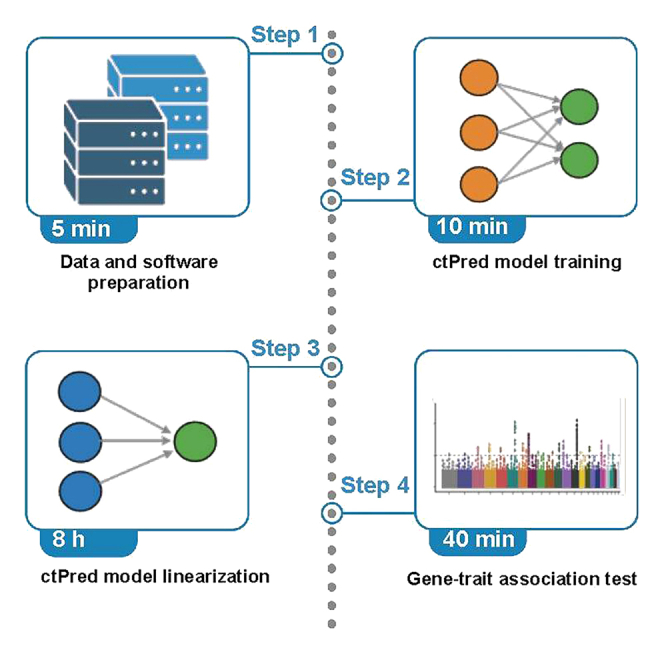

## Before you begin

This protocol describes the specific steps for performing cell-type–specific transcriptome-wide association studies (TWASs) using the **scPrediXcan** framework.^1^ scPrediXcan is a TWAS framework that integrates single-cell data using deep learning, enabling discovery of cell-type–specific genetic effects on disease. It leverages Enformer,^2^ a deep learning sequence-to-epigenome model, as a feature extractor to train ctPred, which predicts cell-type–specific expression from genotype at the individual level. A linearized implementation of ctPred reduces the computational burden to provide population-level scalability without compromising performance.

**Figure 1:**
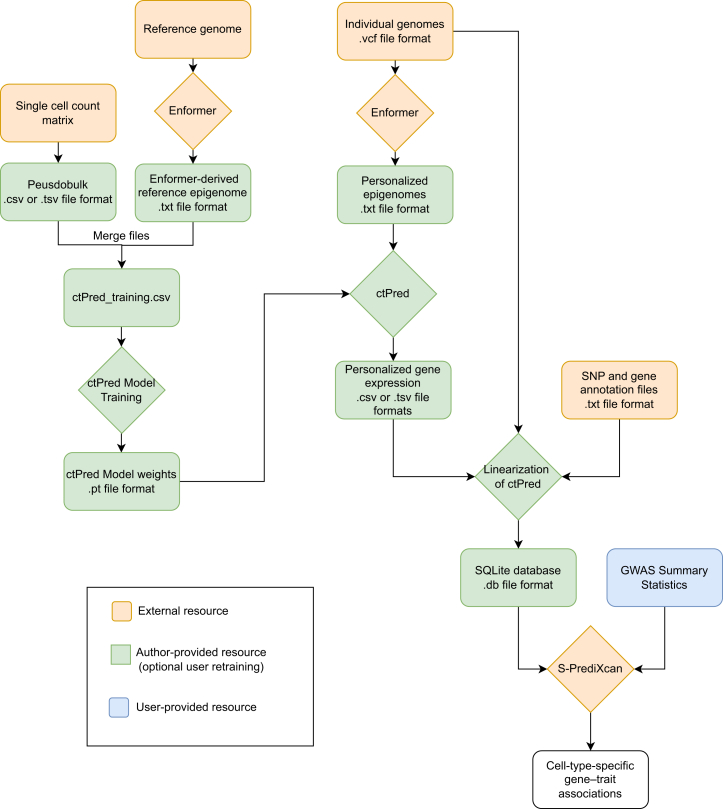
Workflow for scPrediXan training Steps are color coded by the source of the data/resource: external (yellow), author-provided (green), and user-provided (blue).

The detailed procedure includes one step for data/software preparation followed by three main phases of data processing: (1) using paired epigenomic features and pseudoubulk expressions of the gene to generate cell-type–specific expression models (ctPred) that predict expression from individual genotypes; (2) linearizing the trained models into elastic net form (ℓ-ctPred) to provide summary statistics for the association test in the next step; and (3) performing association testing between candidate causal genes and phenotypes with S-PrediXcan.^3^ A schematic of the full workflow, including key files, is provided in Figure 1. This protocol relies on Python scripts. The complete set of scripts is available in the scPrediXcan repository (see key resources table).

We provide pretrained ℓ-ctPred models (46 cell types in total^1^), which users can directly apply to GWAS phenotypes that are likely associated with immune cell types (e.g., memory B cells) or pancreatic islet cell types to perform trait association tests. For other phenotypes, users are encouraged to train their own models to ensure accurate, cell-type–specific predictions.

The scPrediXcan approach is trained with European ancestries but supports TWAS analyses in non-European and admixed ancestries. To apply the framework to another ancestral population, users should follow the retraining procedure using ancestry-matched data. Similarly, although our framework is designed for human TWASs, it is also applicable to mice by following this full protocol using corresponding murine data and employing the prediction head for murine epigenomic features in the Enformer model.

### Innovation

The scPrediXcan framework introduces several conceptual and technical innovations to enable single-cell TWASs. Our use of Enformer as a biologically informed feature extractor provides a mechanistic bridge between genetic sequence, regulatory activity, and gene expression. By pairing the epigenomic predictions with cell-type–specific gene expression data, the ctPred model achieves cell-type resolution. The linearization of ctPred model maintains predictive performance and resolution, while enabling population-level TWASs. While scPrediXcan comes fully trained for human immune and pancreatic cells, its modular design allows for user-directed training for broad application across additional cell types, ancestries, and species.

### Software preparation

**Timing: 10–20 min**

scPrediXcan requires a Linux or MacOS system with a minimum 16 RAM available; for full training, 64 RAM is suggested. It also requires Conda, Python, Nextflow, and the deep learning package Pytorch. This step outlines how to create a Conda environment (named scPrediXcan) and install the software packages needed (above) on Linux or MacOS. The user must install and initialize Conda before the first step.

1. For Linux: Run the following code in the terminal to create a parent folder named scTWAS for all downloaded data and generated results. It will also create a new environment in Conda with the and activate the environment for running scPrediXcan.

# Make project parent file

mkdir scTWAS

cd scTWAS

# Clone repositories for ctPred training

git clone https://github.com/hakyimlab/scPrediXcan.git

# Create and activate conda environment for Linux

conda env create -f scPrediXcan/scPrediXcan_env.yml

conda activate scPrediXcan

***Alternatives*:** For MacOS, use -f scPrediXcan/scPrediXcan_mac_env.yml for conda environment creation.

# Make project parent file

mkdir scTWAS

cd scTWAS

# Clone repositories for ctPred training

git clone https://github.com/hakyimlab/scPrediXcan.git

# Create and activate conda environment for MacOS

conda env create -f scPrediXcan/scPrediXcan_mac_env.yml

conda activate scPrediXcan

2. Clone the PredictDb-nextflow and S-PrediXcan^3^ repositories and create a folder to store the results.

# Clone repositories for l-ctPred training and S-PrediXcan

git clone https://github.com/hakyimlab/PredictDb-nextflow.git

git clone https://github.com/hakyimlab/MetaXcan

mkdir Results

***Note:*** At the end of Software preparation, you should have a parent folder named scTWAS on your local hard drive that contains four subfolders: scPrediXcan, PredictDb-nextflow, MetaXcan, and Results.

### Dataset preparation

**Timing: 5 min to 4 h**

The data and software described below will be needed for training cell-type–specific ctPred models that predict gene expression levels from Enformer-derived epigenomic features. Enformer is a preexisting deep learning model that predicts epigenomic features from a reference genome. The Enformer-derived epigenomic features are used to train ctPred models. Users can use toy or real example data (see key resources table) to test the training steps. The toy example data is limited to one cell type (Acinar), ∼20,000 genes and 6 individuals (ct-Pred training) or 224 genes and 20 individuals (ℓ -ctPred training) to provide users with an end-to-end tutorial that can be completed in <4 hours and requires less memory (16 GB). Data download is the rate limiting step, with the remainder of the tutorial pipeline taking ∼30 minutes to run. Real example data may take >10 hours, not including data downloads, and requires 32GB RAM.

3. If using toy example data, use the following code to download the data from Zenodo. Using this data, users can run all components of the pipeline with minimal computational burden.

# Download the all the data from Zenodo

zenodo_get 18124207

tar -xzf Download_dataset.tar.gz && rm Download_dataset.tar.gz

***Note:*** This step creates a folder scTWAS/Download_dataset containing the example files. This process may take several hours. If the download fails, download and extract the data manually here: scPrediXcan toy example data: https://zenodo.org/records/18124207. The default file path is /scTWAS/Download_dataset.

4. If using user-provided single-cell RNA-seq (scRNA-seq) data or the example real data (see key resources table), process the count cell-by-gene count matrix into pseudobulk individual-by-gene format for each cell type by aggregating the cells from the same cell type.
  a. For quality control, filter the count matrix to include only cells expressing more than 200 gene features, with each included gene feature present in at least three cells.
  b. Remove doublets and triplets using DoubletDecon.^4^
  c. Remove non-coding sequences by filtering out genes that are absent in the Enformer-derived epigenomic features file we provide (see key resources table).
  d. Split the matrix by cell types using cell-type labels, generating one individual-by-gene matrix per cell type.
  e. Aggregate counts across all cells belonging to the same individual for each cell type, converting the cell-by-gene count matrix into an individual-by-gene count matrix.

***Note:*** The epigenomic predictions provided were derived by Enformer^2^ for protein-coding genes available in BioMart. The gene identifiers must be consistent across the scRNA-seq matrix and Enformer feature file. Users can use the gene names to align the files if gene IDs are from different versions.

***Note:*** Removing doublets and triplets is a standard quality control procedure for scRNA-seq data to avoid artificial expression profiles that can distort downstream analyses. Tools such as DoubletDecon detect and remove these multiplets by identifying expression profiles that appear as mixtures of distinct cell types, thereby preserving the integrity and biological interpretability of downstream analyses in scPrediXcan.

5. Load the count matrix prepared above and the Enformer-derived epigenomics features file. Make sure that the column name for gene names is ‘gene_name’ for both files, so that they can be merged according to this column.
6. Run the following Python script. The code will complete the following steps:
  a. Calculate mean expression across individuals.
  b. Convert mean expression to rank-based percentiles.
  c. Merge mean expression percentiles with the epigenomics file to create a single .csv file (genes as rows and 5316 columns: generic index ID, gene name, 5313 columns for epigenomic features, and expression percentile).
  d. The output file will be saved in scTWAS folder and will serve as the training file for ctPred.

**CRITICAL:** All code beyond this point is given using file paths created using the example data. Users training with user-provided data should replace the file paths as appropriate.

python scPrediXcan/Scripts/ctPred/data_prep.py ∖

 --exp './Download_dataset/Acinar_normcounts.csv' ∖

 --epi './Download_dataset/Gene_epigenomics_v0.csv' ∖

 --output './Download_dataset/training_cell.csv'

***Note:*** The saved training_cell object is a .csv file in the ./Download_dataset folder. This file will serve as the input data for ctPred training. ctPred is trained to predict expression percentiles rather than raw counts because the distribution of gene expression across individuals is highly skewed; using quantiles stabilizes training.

## Key resources table

**Table:** 

REAGENT or RESOURCE	SOURCE	IDENTIFIER
**Software and algorithms**		

scPrediXcan	Zhou et al.^1^	https://github.com/hakyimlab/scPrediXcan
PrediXcan	Gamazon et al.^5^	https://github.com/hakyimlab/PrediXcan
S-PrediXcan	Barbeira et al.^3^	https://github.com/hakyimlab/MetaXcan
Enformer	Avsec et al.^2^	https://github.com/google-deepmind/deepmind-research/tree/master/enformer
DoubletDecon	DePasquale et al.^4^	https://www.sciencedirect.com/science/article/pii/S2211124719312860

**Deposited data**		

Toy example data: 19563 genes, 6 individuals (ctPred training) or 224 genes, 20 individuals (ℓ -ctPred training), Acinar cells	Zhou et al.^1^	scPrediXcan toy example data: https://doi.org/10.5281/zenodo.18124207
Real example data: Enformer-derived epigenomic features	Zhou et al.^1^	scPrediXcan real example data: https://doi.org/10.5281/zenodo.17544851
Real example data: Geuvadis individual genotype data from 1000G project	The 1000 Genomes Project Consortium^6^	Guevadis genotype data: https://doi.org/10.5281/zenodo.16538482
Pre-trained ℓ -ctPred weights for islet cells	Zhou et al.^1^	*ℓ*-ctPred islet cell data: https://doi.org/10.5281/zenodo.15318900
Pre-trained ℓ -ctPred weights for immune cells	Zhou et al.^1^	*ℓ*-ctPred immune cell data: https://doi.org/10.5281/zenodo.14346661
User-provided data		
(Optional) scRNA-seq count matrix for training ctPred	Variable	Variable sources. File format typically .mtx, .tsv, .csv, or .h5. Example data can be found here: scPrediXcan real example data: https://doi.org/10.5281/zenodo.17544851
(Optional) Alternative individual genotype data for ctPred linearization	Variable	Variable source. File format typically .vcf
GWAS or meta-analysis summary data for association test	Variable	Variable sources. File format typically .txt.gz Example data can be found here: scPrediXcan real example data: https://doi.org/10.5281/zenodo.17544851

**Other**		

GWAS summary statistics harmonization pipeline	Barbeira et al.^7^	GWAS Harmonization And Imputation · hakyimlab/summary-gwas-imputation Wiki (github.com)

## Step-by-step method details

### Training the ctPred model for gene expression prediction from epigenomic features

**Timing: 5–8 min per cell type**

ctPred is a multilayer perceptron to predict gene expressions at pseudobulk level from gene epigenomic representations (i.e., Enformer-output epigenomic features).

1. Train ctPred for each cell type replacing “training_cell.csv” with the path containing the appropriate training files in .csv format.
  a. Input: Merged file (.csv format) containing population average gene expression and gene epigenomic features (see data preparation).
  b. Before running the code, please open and modify the ctPred_train.json file variables with your file paths.
    ***Note:*** The training file for each cell type must contain a gene-by-epigenomics matrix concatenated with a gene by expression percentile matrix (.csv format with genes as rows and 5316 columns: generic index ID, gene name, 5313 columns for epigenomic features, and expression percentile).
    ***Note:*** Training for all cell types can be run in parallel to reduce training time.
  c. Output: ctPred model weights file in .pt format.
    ***Note:*** The trained model in .pt format will be generated in the ./Results folder.
    # Train ctPred and save the results
    python scPrediXcan/Scripts/ctPred/ctPred_train.py ∖
     --parameters 'scPrediXcan/Scripts/ctPred/ctPred_train.json' ∖
     --cell_file './Download_dataset/training_cell.csv'

### Linearizing ctPred for gene expression prediction from genotype dosage

**Timing: 3–10 h**

This section describes ℓ-ctPred training. The ℓ-ctPred models infer a linear relationship between genotype data and the ctPred-predicted expression percentiles. We provide the appropriate code below each minor step.

2. Clone the PredictDb-nextflow repository. If completed in Software preparation, skip this step.

# Clone repository

git clone https://github.com/hakyimlab/PredictDb-nextflow.git

3. Use ctPred models from above to predict the personalized gene expressions from personalized epigenomic features. Matrices should be gene-by-epigenome with one matrix per individual. Concatenate expression for each individual into one file.
  a. Input: Personalized epigenomic features, obtained from Enformer (.h5 file format, containing 4 central bins 5313 epigenomic features matrix for each person) and ctPred model (.pt file format).
  b. Output: Personalized gene expression in a .csv file format.

# Personalized prediction

python scPrediXcan/Scripts/ctPred/predict_h5.py ∖

 --model_path './Results/training_cell_ctPred.pt' ∖

 --input_folder 'Download_dataset/Personalized_epigenomics' ∖

 --ref_cs

'Download_dataset/coding_gene_biomart_TSS_dictionary.csv' ∖

 --output_path './Results/Personalized_predictions.csv'

***Note:*** Personalized predictions will be saved in the./Results folder.

4. Run the PredictDb nextflow pipeline.
  a. Input: Genotype data file in .vcf format (i.e., Geuvadis individuals genotype data from 1000G program or user-provided). ctPred-predicted gene expression file in .csv or .tsv format for each cell type. Gene annotation file and SNP annotation file both in .txt format. These are created using the genotype data and expression data above according to PredictDb tutorial (PredictDb-nextflow/docs/usage.md at master · hakyimlab/PredictDb-nextflow (github.com)).
  b. Output: Transcriptome model SQLite database (i.e., ℓ-ctPred) and a SNP covariance matrix file for each cell type.

***Note:*** The toy example dataset used in this step includes 20 individuals with precomputed personalized expression for 224 genes in Acinar cells.

***Note:*** The gene and SNP annotation files should both contain annotations for all genes on chromosomes 1–22 and the sex chromosomes.

**CRITICAL:** The genotype file must contain the dosage of each sample for the specific varID with samples in columns and varID as rows.

**CRITICAL:** The file 'ctPred_predicted_gene_expression.csv’ must contain the ctPred-predicted individual gene expressions according to the individual epigenomic features pre-calculated by Enformer. This is created in **(3)**.

***Alternatives:*** We provide genotype data from 448 Geuvadis individuals (see key resources table) along with ctPred-predicted gene expression profiles to fit an elastic-net model for each cell type, but alternative genotype reference panels can also be used at this stage.

Code using toy example data:

# Run PredictDb nextflow pipeline with example data

 nextflow run ./PredictDb-nextflow/main.nf ∖

 -profile local ∖

 --gene_annotation './Download_dataset/Gene_anno.txt' ∖

 --snp_annotation './Download_dataset/T2D_snp_anno_v3.txt' ∖

 --genotype './Download_dataset/T2D_dosage_v3.txt' ∖

 --gene_exp './Download_dataset/Acinar_train_v0.csv' ∖

 --outdir ./Results ∖

 --keepIntermediate ∖

 --nfolds 3 ∖

 -resume

***Note:*** Because of the limited sample size in the toy example (i.e., 20 individuals), this step may occasionally fail when one or two genes have constant expression values within a batch. Such failures are rare but can be resolved in most cases by deleting the ./pipeline_info folder (see Alternatives below) and reattempting the training. In real-world applications, where expression data from more than 100 individuals are typically available, this issue does not arise.

**CRITICAL:** Please use the next code cell when training with user-provided data, which uses the default 10-folds for cross-validation. Due to the small sample size of the toy example and the use of random seeds in the PredictDb pipeline, we explicitly set the number of cross-validation folds to 3 via the nfolds argument to ensure that each fold contains a sufficient number of samples. Users may omit this argument, in which case the default 10-folds will be used.

Template code for training with user-provided data:

# Replace the file paths with your own here

nextflow run ./PredictDb-nextflow/main.nf ∖

 --gene_annotation 'Gene_anno.txt' ∖

 --snp_annotation 'snp_annot.txt' ∖

 --genotype 'genotype_file' ∖

 --gene_exp 'ctPred_predicted_gene_expression.csv' ∖

 --outdir results_path ∖

 --keepIntermediate ∖

 -resume

***Note:*** The template code for training with user-provided data requires 32 GB RAM. If using hardware with less RAM available, please enable a low memory profile (requires 16 GB RAM) by replacing the first line of code with these two lines. This will significantly increase the training time.

# Run PredictDb nextflow pipeline with low memory

nextflow run ./PredictDb-nextflow/main.nf ∖

 -profile local ∖

***Alternatives:*** If for any reason, the PredictDb nextflow pipeline fails—indicated by the absence of the filtered_db folder in the ./Results directory—please run the code below to delete the ./pipeline_info folder. Re-run the PredictDb nextflow pipeline.

# If PredictDb nextflow fails, run this line before reattempting

rm -r ./Results/pipeline_info

### Testing gene-trait association to prioritize candidate causal genes

**Timing: 30–80 min**

This section describes the implementation of Summary-PrediXcan (S-PrediXcan),^3^ the gene–trait association step between user-provided GWAS data and expression levels obtained from ℓ-ctPred models.

5. Clone the S-PrediXcan repository and go to the software folder. If completed in Software preparation, skip this step.

# Clone repository and enter software directory

git clone https://github.com/hakyimlab/MetaXcan

6. Run the S-PrediXcan script.
  a. Input: User-provided GWAS or meta-analysis summary data (.txt file format) and ℓ-ctPred sqlite database (.db file format) from (**4b)** for each cell type. Users can also use the provided pretrained ℓ-ctPred files (islet and immune cells; see key resources table). To make the GWAS summary statistics compatible with the S-PrediXcan method, users can follow the GWAS data harmonization method here: GWAS Harmonization And Imputation · hakyimlab/summary-gwas-imputation Wiki (github.com).^7^
    ***Note:*** The variables used in the code below (‘panel_variant_id’, ‘effect_allele’, ‘non_effect_allele’, ‘effect_size’, and ‘pvalue’) refer to the columns of the GWAS summary statistics file. Respectively, these variables represent the SNP ID, the reference allele, the alternative allele, the effect size, and the p value. Modify these variable names according to the column names in your file.
    **CRITICAL:** scPrediXcan performs the association testing using the S-PrediXcan method. Currently, S-PrediXcan supports GWAS summary statistics that report effect sizes (β coefficients). GWAS summary statistics reported in terms of odds ratios (ORs) are not directly compatible, as conversion from OR to β can vary depending on the GWAS model, sample ascertainment, and trait type.
  b. Output: A file in .csv format containing the standard TWAS summary statistics for each cell type.
    ***Note:*** While the toy ℓ-ctPred model can be used in the following code, it includes a limited number of genes and therefore does not produce meaningful TWAS results (0% of SNPs used). Therefore, to demonstrate the S-PrediXcan step, we present the code using a fully trained ℓ-ctPred model for one cell type (i.e., beta cells, ∼20,000 genes, 448 individuals, file name: scPrediXcan_Beta_T2D.db). This S-PrediXcan implementation uses ∼86% of SNPs.
    # Run S-PrediXcan using l-ctPred models and GWAS summary statistics
    python3 ./MetaXcan/software/SPrediXcan.py ∖
     --model_db_path './Download_dataset/scPrediXcan_Beta_T2D.db' ∖
     --model_db_snp_key varID ∖
     --covariance
    './Download_dataset/predict_db_Model_training_filtered.txt' ∖
     --gwas_file './Download_dataset/T2D_harmonized.txt.gz' ∖
     --snp_column panel_variant_id ∖
     --effect_allele_column effect_allele ∖
     --non_effect_allele_column non_effect_allele ∖
     --beta_column effect_size ∖
     --pvalue_column pvalue ∖
     --gwas_N 492191 ∖
     --gwas_h2 0.1083 ∖
     --keep_non_rsid ∖
     --output_file './Results/S_prediXcan_Beta.csv'

## Expected outcomes

The final output of this protocol should be a file in .csv format containing the standard TWAS summary statistics between the BioMart genes and the complex trait in from the user-provided GWAS. The data will be arranged in columns, comprising gene id, gene name, z-score, effect size and p value. See Table 1 for an example.

**Table 1:** Example of scPrediXcan final output

gene	gene_name	Zscore	effect_size	Pvalue
ENSG00000165066	NKX6-3	−14.297	−48.6426	2.29E-46
ENSG00000153814	JAZF1	−13.0641	−45.1497	5.28E-39
ENSG00000125746	EML2	−13.016	−14.659	9.92E-39
ENSG00000151465	CDC123	−12.9137	−34.8207	3.77E-38
ENSG00000109501	WFS1	12.45714	12.23656	1.28E-35
ENSG00000090316	MAEA	−12.3646	−22.0724	4.06E-35
ENSG00000073792	IGF2BP2	−12.32	−120.023	7.07E-35
ENSG00000165609	NUDT5	−12.0674	−71.3792	1.57E-33

## Limitations

scPrediXcan offers a robust framework for performing cell-type–specific TWASs using predicted gene expression levels derived from genetic sequence features. However, there are important limitations to consider.

First, ctPred—the model used to predict gene expression—relies on Enformer as its sequence encoder. While Enformer is effective in capturing regulatory signals from DNA sequences, some of its predictions may show negative correlation with measured expression levels. This is a challenge shared by current sequence-based models trained on the reference genome. Consequently, this protocol emphasizes statistical significance of correlation (i.e., p values) rather than direction, which limits our ability to interpret whether increased disease risk is associated with up- or downregulation of a specific gene—an important factor in therapeutic development.

Second, like most TWAS frameworks, scPrediXcan primarily models cis-regulatory effects and does not account for trans-regulatory interactions, which could impact predictions in a cell-type–specific context. Additionally, linkage disequilibrium structure can introduce confounding signals in association results, a common issue in summary-statistics–based methods.

Finally, the genetic reference panels and GWAS datasets commonly used with scPrediXcan are predominantly based on individuals of European ancestry. This may reduce the method’s performance when it is applied to other populations. We encourage users to construct and utilize ancestry-matched reference datasets, which is fully supported by the provided tools.

## Troubleshooting

### Problem 1

The genes in the scRNA-seq dataset do not correspond exactly with the protein-coding genes from BioMart, for which we provide the Enformer-predicted epigenomic features.

### Potential solution

By default, the ctPred training process includes only protein-coding genes as defined by BioMart. Users should ensure that their input gene set corresponds to those genes for which epigenomic features are available. For genes included in this list but not expressed in the observed data, expression values should be set to zero prior to computing the population-level mean and subsequent percentiles. We set it as the standard data process procedure in the Data preparation step.

Genes not included in the Biomart protein-coding list, but expressed in the data, are removed automatically. Since the Biomart protein-coding gene list covers almost all protein-coding genes, the removal is generally trivial. However, if a substantial number of Biomart protein-coding genes are missing in the scRNA-seq data, this may indicate low data quality. To help users monitor this, a line of code has been added to report the proportion of genes with zero counts. If this proportion is unusually high, the user should evaluate their data quality more closely.

If users wish to include additional gene types—such as mitochondrial genes—that are not part of the default set, they must first use Enformer to predict the corresponding epigenomic features from reference genome sequences. Once the epigenomic features are obtained, users can include those genes and proceed with the standard ctPred training workflow.

### Problem 2

The user-provided GWAS summary statistics file is not immediately compatible with the format required for (**6**), where gene–trait association testing is performed using S-PrediXcan, or there are discrepancies between the genome build used in the GWAS summary statistics and the genotype reference panel from Data preparation. Inconsistencies in genome builds or column naming conventions can prevent proper alignment with the variant definition in the genotype reference panel. If this occurs, the final report of S-PrediXcan will give the following as the last line: ‘INFO - 0 % of model’s snps used’.

This is more likely to occur with older GWAS datasets, which may be aligned to older genome builds (e.g., hg18), whereas the genotype reference panel we use in PrediXcan is based on a more recent build (i.e., GRCh38).

### Potential solution

Convert the GWAS summary statistics to the appropriate genome build to ensure matching formats. Follow the provided script and instructions to perform file harmonization: (GWAS Harmonization And Imputation · hakyimlab/summary-gwas-imputation Wiki (github.com)).^7^ Once harmonized, the GWAS summary statistics can be used in the downstream stages of the pipeline.

### Problem 3

scPrediXcan reports p values for some genes that are not expressed in the target cell type.

### Potential solution

By default, ctPred predicts the expression level of any gene using Enformer-derived epigenomic features. While the training procedure encourages ctPred to assign low expression values to genes that are lowly or not expressed, it may still predict nonzero expression for some truly unexpressed genes. This can occur because ctPred groups truly unexpressed genes, genes expressed at a low level that are undetected due to limited sequencing depth, and unmeasured genes together. This grouping makes it difficult for the model to perfectly distinguish truly unexpressed genes from those with very low expression. We recommend that users filter out genes not expressed in the target cell type before interpreting or reporting scPrediXcan results.

## Resource availability

### Lead contact

Requests for further information and resources should be directed to and will be fulfilled by the lead contact, Hae Kyung Im (haky@uchicago.edu).

### Technical contact

Specific questions about technical aspects of the protocol should be directed to and will be answered by the technical contact, Yichao Zhou (yichaozhou@uchicago.edu).

### Materials availability

This study did not generate any new or unique reagents.

### Data and code availability

• All data (including example data and author-generated metadata) required to fully train our published framework have been deposited at Zenodo as scPrediXcan real example data: https://doi.org/10.5281/zenodo.17544851, scPrediXcan toy example data: https://doi.org/10.5281/zenodo.18124207 and are publicly available as of the date of publication. All original code has been deposited at GitHub and is publicly available at https://github.com/hakyimlab/scPrediXcan as of the date of publication.
• Any additional information required to reanalyze the data reported in the paper is available from the lead contact upon request.

## Acknowledgments

We thank BioRender.com for providing the platform used to edit the graphical abstract. This research used resources of the Argonne Leadership Computing Facility, a DOE Office of Science User Facility supported under contract DE-AC02-06CH11357. This work was completed in part with resources provided by the University of Chicago’s Research Computing Center and Beagle3. We also acknowledge resources from the Center for Research Informatics, funded by the Biological Sciences Division at the University of Chicago, with additional funding provided by the Institute for Translational Medicine, CTSA grant 2U54TR002389-06 from the National Institutes of Health. Y.Z., M.C., and H.K.I. were partially funded by R01 GM126553 and R01 HG011883. Y.Z., S.S.-M., T.A., and H.K.I. were funded in part by R01AA029688. H.K.I. was funded in part by P30DK020595.

## Author contributions

Conceptualization, Y.Z., M.C., and H.K.I.; methodology, Y.Z., T.A., S.G., F.N., and H.K.I.; software, Y.Z., T.A., S.G., F.N., and H.K.I.; formal analysis, Y.Z. and H.K.I.; data curation, Y.Z. and F.N.; visualization, Y.Z. and S.S.-M.; resources, M.C., R.M., R.K., H.K., J.P., and H.K.I.; protocol development and testing, Y.Z. and S.S.; writing – original draft, Y.Z. and S.S.; writing – review and editing, Y.Z., S.S., S.S.-M., B.L., M.C., and H.K.I.; funding acquisition, M.C. and H.K.I.

## Declaration of interests

The authors declare no competing interests.
